# Dual role of ACE2 in regulating inflammation triggered by Omicron S1 and other SARS-CoV-2 Spike variants

**DOI:** 10.3389/fimmu.2025.1667880

**Published:** 2026-01-06

**Authors:** Annamaria Pedoto, Juan M. Lozano-Gil, María Ocaña-Esparza, Ana M. Conesa-Hernández, Sergio Candel, María L. Cayuela, Victoriano Mulero, Sylwia D. Tyrkalska

**Affiliations:** 1 Departamento de Biología Celular e Histología, Facultad de Biología, Universidad de Murcia, Murcia, Spain; 2 Instituto Murciano de Investigación Biosanitaria Pascual Parrilla (IMIB)-Arrixaca, Murcia, Spain; 3 Centro de Investigación Biomédica en Red de Enfermedades Raras (CIBERER), Instituto de Salud Carlos III, Madrid, Spain

**Keywords:** ACE2, COVID-19, Omicron variant, SARS-CoV-2, Spike protein, zebrafish

## Abstract

Since the emergence of SARS-CoV-2 in late 2019, substantial efforts have been made to understand its mechanisms of pathogenicity. Although angiotensin-converting enzyme 2 (ACE2) has been identified as the main receptor for viral entry, the complexity of the host immune response to different Spike protein conformations and variants remains poorly understood. Using zebrafish larvae as an *in vivo* model, we show that the monomeric S1 domain of the Omicron variant triggers a potent proinflammatory response characterized by elevated Nfkb activity and increased expression of key cytokines, despite reduced recruitment and expansion of neutrophils and macrophages. Notably, monomeric S1 Omicron also promotes neutrophil cell death, suggesting an alternative mechanism of immune modulation. In contrast, the trimeric form of the Spike protein fails to induce significant inflammation or emergency hematopoiesis, likely due to its efficient neutralization by endogenous Ace2. Our results revealed that both zebrafish and human ACE2 exert a dual anti-inflammatory role: indirectly through the production of angiotensin-(1-7), and directly by binding and neutralizing the trimeric Spike. These results provide new insights into variant-specific immune responses and the multifaceted role of ACE2 in modulating SARS-CoV-2-induced cytokine storm syndrome.

## Introduction

Since the beginning of the COVID-19 pandemic in late 2019, extensive research has been conducted on this disease and its causative agent, severe acute respiratory syndrome coronavirus 2 (SARS-CoV-2). Despite significant progress, many aspects of this virus’s pathogenicity and mechanisms of action remain poorly understood (1). A notable case of a key question still unresolved is the molecular and pathogenic consequences of the interaction between the receptors that facilitate direct viral interaction with host cells and mediate viral entry (2, 3). Numerous studies have shown that human angiotensin-converting enzyme 2 (hACE2) acts as the primary receptor for SARS-CoV-2, binding to the viral spike protein (S) and allowing the virus to internalize into the host cell (4–8).

SARS-CoV-2 is a single-stranded, positive-sense RNA virus enclosed within a lipid envelope, and it encodes four structural proteins: envelope (E), membrane (M), nucleocapsid (N), S, the latter being the largest and most functionally significant (9, 10). From an immunological perspective, the S protein is the most immunogenic component of the virus. It assembles into trimers, forming large protrusions on the viral surface, which give the virus its characteristic “spike” appearance (11). The S protein consists of two distinct domains, S1 and S2, each with a specific role in viral entry. The S1 domain mediates initial interaction with receptor molecules on the host cell surface, while the S2 domain facilitates membrane fusion between the viral envelope and the host cell, enabling the release of the viral genome into the cytoplasm (12). Notably, within the S1 domain, the receptor-binding domain (RBD) exhibits high affinity for ACE2, binding strongly not only to the membrane-bound receptor but also to its soluble forms (2, 5, 7). Additionally, several host factors have been implicated in facilitating viral entry and membrane fusion. These include transmembrane proteases such as disintegrin and metalloprotease domain 17 (ADAM17), transmembrane protease serine 2 (TMPRSS2), and TNF-converting enzyme, as well as structural proteins like vimentin and clathrin, which may enhance viral binding and internalization (13–17).

An important consideration in the interaction between SARS-CoV-2 and ACE2 is the structural organization of the S protein. In its native state, S proteins assemble into trimers, forming club-shaped protrusions on the viral membrane surface. These trimeric structures are primarily responsible for host cell entry and membrane fusion (18). Spike trimers can adopt multiple conformational states, including the closed, open, and intermediate forms characterized by mobile but predominantly closed receptor-binding domains (RBDs). In the open conformation, the RBD of one or two monomers becomes exposed, allowing for ACE2 recognition and binding. Conversely, in the closed conformation, all three RBDs remain shielded by the N-terminal domain (NTD) of the S protein, limiting receptor accessibility (19, 20). Despite the established role of trimeric S in viral entry, it remains unclear whether monomeric S fragments, which may circulate in infected and vaccinated individuals, can elicit similar cellular effects and immune responses in the host (21–24). Therefore, further studies are needed to determine whether S monomers contribute to pathogenesis *in vivo*.

ACE2 functions both as an enzyme and as a critical receptor expressed on the surface of various cell types. It is most abundant in the gut, followed by the kidney, while its expression in the lung and heart is much lower. ACE2 can also be shed into the plasma, where it exists in two distinct forms: a membrane-bound (insoluble) form and a circulating soluble form, however the circulating ACE2 levels are very low to non-detectable, particularly in comparison to ACE (25–28). ACE2 plays a key role in the renin–angiotensin–aldosterone system (RAAS), a homeostatic regulator of vascular function (29). Within this system, renin, primarily produced by the kidneys, cleaves angiotensinogen derived from the liver to generate angiotensin I (Ang I). Subsequently, ACE converts Ang I into Ang II, which exerts its effects by binding to two distinct receptors: Ang II type 1 receptor (AT1R) and Ang II type 2 receptor (AT2R) (30, 31). Depending on the receptor engaged, Ang II can elicit divergent physiological responses. Binding to AT1R promotes vasoconstriction, oxidative stress, inflammation, and fibrosis, whereas activation of AT2R mediates opposing effects, including vasodilation and anti-inflammatory signaling (32, 33). Moreover, ACE2 counterbalances the activity of ACE by degrading Ang II into Ang-(1-7), a peptide with anti-inflammatory, vasodilatory and cardioprotective properties, thereby contributing to blood pressure regulation and cardiovascular homeostasis (34). SARS-CoV-2, particularly its RBD within the S1 subunit of the S protein, competes with Ang II for binding to hACE2 (6, 35). This interaction not only obstructs ACE2 activity but also downregulates its membrane expression, thereby disrupting the balance of the RAAS and contributing to disease pathogenesis (36–38). Interestingly, despite the high-affinity interaction between the viral RBD and ACE2, the catalytic active site of ACE2 remains unoccupied, and this binding occurs independently of the enzyme’s peptidase activity (39).

Since the onset of the COVID-19 pandemic, multiple SARS-CoV-2 variants of concern (VOCs) have emerged, each characterized by distinct mutations, primarily within the S protein (40). These genetic alterations have been associated with variations in disease severity, transmissibility, and immune evasion. The most recent VOC, designated Omicron (lineage B.1.1.529), was first detected in South Africa in November 2021 (41). Importantly, this variant has been reported to replicate approximately 70 times faster than the Delta variant; however, its capacity for deep lung tissue penetration appears to be reduced, correlating with a lower risk of severe disease and hospitalization. Genomic analyses have identified approximately 60 mutations distinguishing Omicron from the ancestral SARS-CoV-2 strain, with 32 of these mutations specifically affecting the S protein (42). Notably, several of these alterations had not been observed in previous VOCs (43). Among these mutations, Omicron exhibits 30 amino acid substitutions, three small deletions, and one small insertion within the S protein, with 15 of these changes localized to the RBD (44). Despite these extensive modifications, it remains unclear which specific mutations underline Omicron’s unique pathogenic and transmissibility characteristics. Further research is needed to determine their functional implications in viral entry, immune escape, and disease progression.

Our laboratory has already developed and validated a successful zebrafish model to study COVID-19 pathogenesis by injecting different variants of the S1 domain from the SARS-CoV-2 S protein into the hindbrain ventricle of zebrafish larva (45–47). In this approach, the larval hindbrain ventricle mimics the microenvironment of human pulmonary alveoli (48), enabling the study of local and systemic immune responses, including COVID-19-associated cytokine storm syndrome (CSS), emergency hematopoiesis, and hemorrhagic events (45, 46). Zebrafish possesses a single copy of the *ace2* gene in its genome, which is highly conserved in both sequence and structure compared to its mammalian counterpart (49). Recent single-cell RNA sequencing analyses have further demonstrated that the RAAS signaling pathway is also evolutionarily conserved between humans and zebrafish (50). In the present study, the zebrafish model has been used to elucidate the dual effect of ACE2 in the pathogenesis of COVID-19-associated CSS using monomeric and trimeric forms of wild-type S1 (S1WT) and to explore the inflammatory properties of Omicron S1. Our findings provide insights into the immunopathogenic mechanisms of SARS-CoV-2 and its emerging variants.

## Results

### Omicron S1 variant is highly proinflammatory

**Figure 1:**
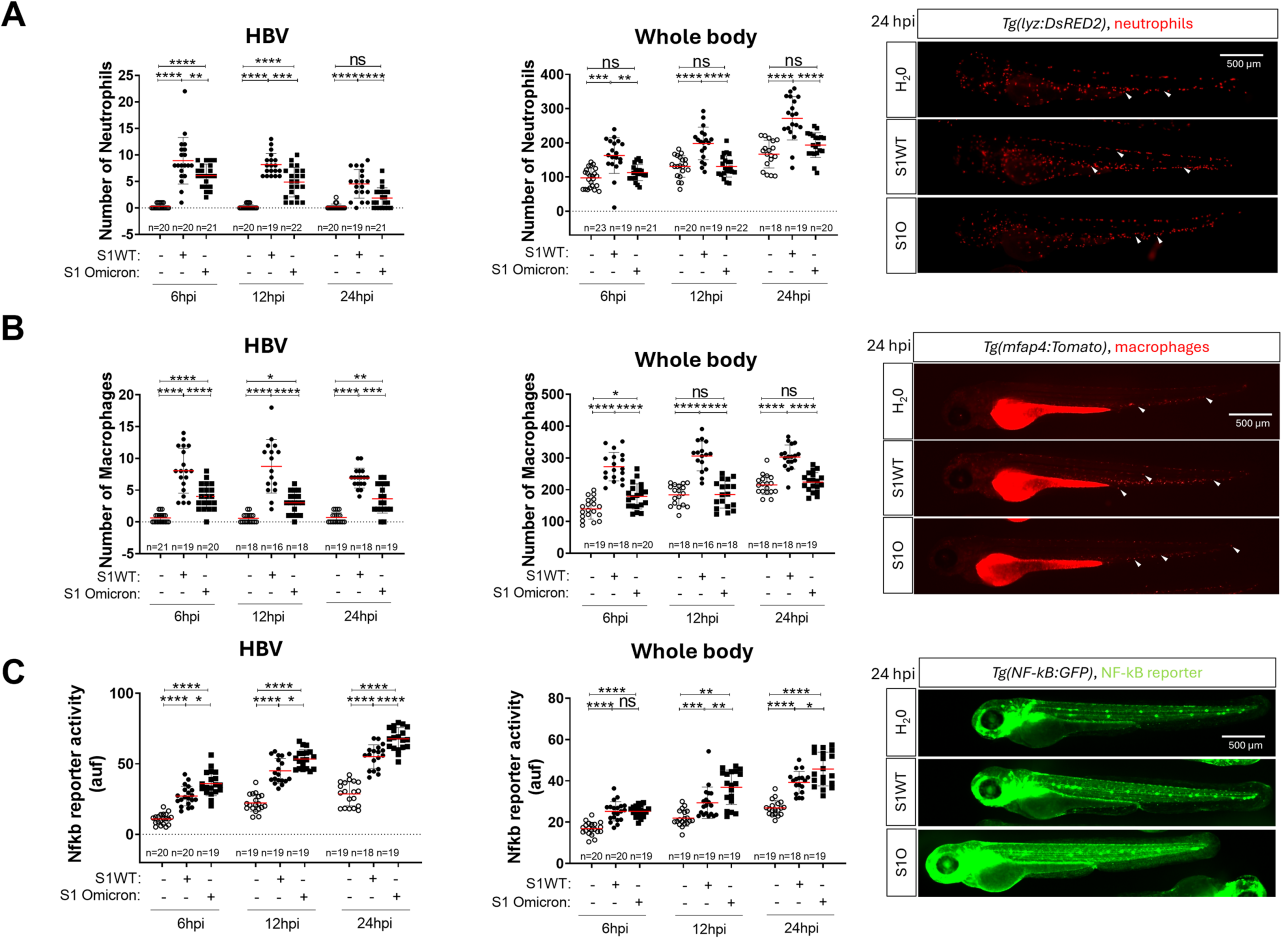
Omicron S1 shows reduced immune cells recruitment and expansion compared with the ancestral variant. Recombinant S1WT, S1 Omicron or vehicle **(-)** were injected in the hindbrain ventricle (HBV) of *Tg(lyz:DsRED)***(A)**, *Tg(mfap4:Tomato)* **(B)**, *Tg(nfkb:eGFP)* **(C)** of 2-dpf larvae. Neutrophil **(A)** and macrophage **(B)** recruitment and number and Nfkb reporter activity **(C)** were analyzed at 6, 12, and/or 24 hpi by fluorescence microscopy. Representative photos for each treatment are shown from 24 hpi. Scale bar 500 μm. Each dot represents one individual, and the means ± SEM for each group is also shown. *P* values were calculated using one-way analysis of variance (ANOVA) and Tukey multiple range test. ns, not significant; *P ≤ 0.05, **P ≤ 0.01, ***P ≤ 0.001 and ****p < 0.0001. auf, arbitrary units of fluorescence.

**Figure 2:**
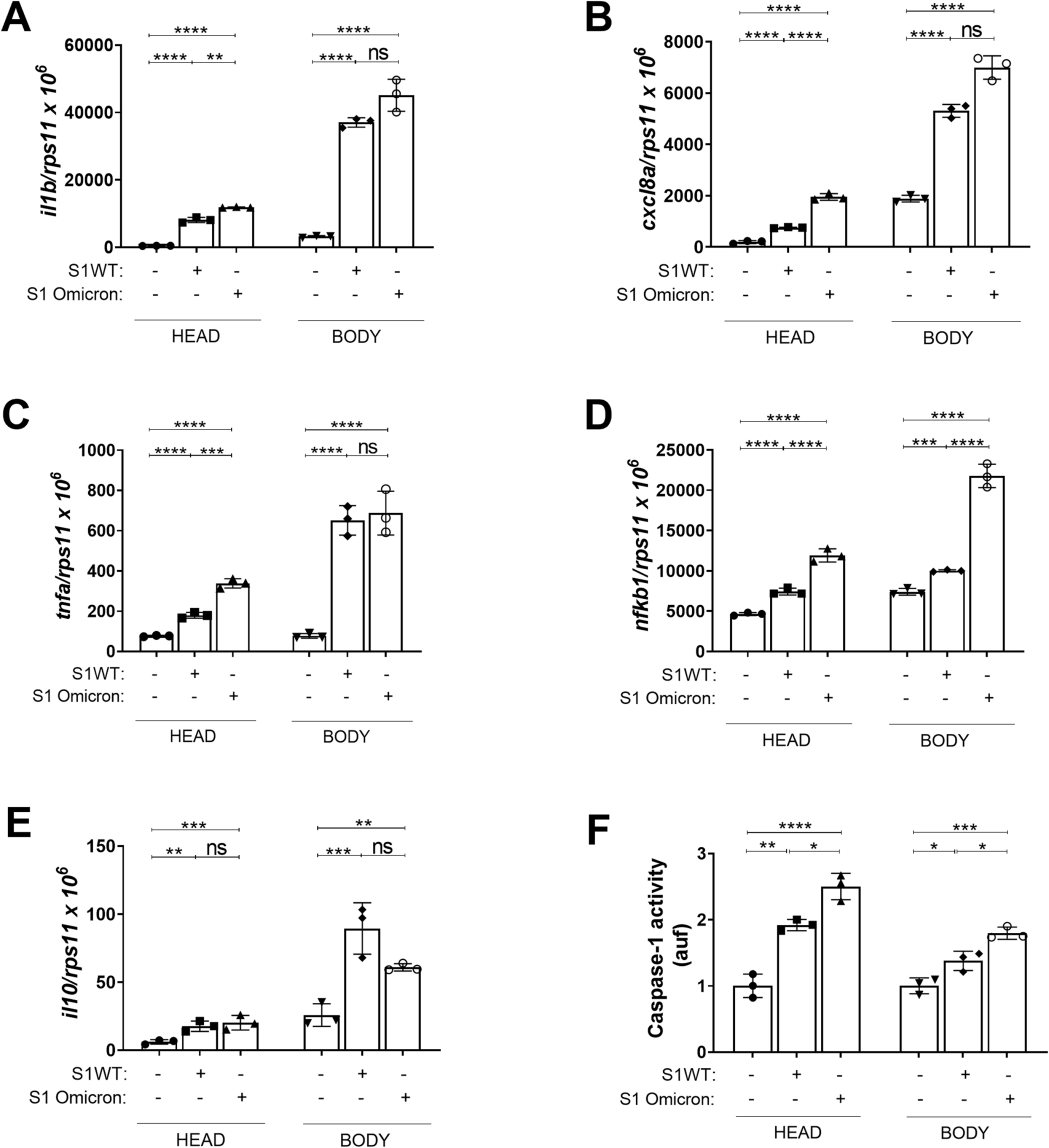
Omicron is more proinflammatory than the ancestral variant. Recombinant S1WT, S1 Omicron or vehicle (-) were injected in the hindbrain ventricle (HBV) of WT **(A–F)** 2-dpf larvae. The transcript levels of the indicated genes **(A–E)** were analyzed at 12 hpi by RT-qPCR in larval head and rest of the body and caspase-1 activity was determined at 24 hpi using a fluorogenic substrate **(F)**. Graphs shown are representative of three independent experiments; technical replicates are displayed in each graph. The means ± SEM for each group is shown. *P* values were calculated using one-way analysis of variance (ANOVA) and Tukey multiple range test. n=45 in **(A–E)**, n=35 in **(F)**. ns, not significant; *P ≤ 0.05, **P ≤ 0.01, ***P ≤ 0.001 and ****p < 0.0001. auf, arbitrary units of fluorescence.

Omicron, one of the latest variants of SARS-CoV-2, presents different, milder symptoms compared to other variants identified to date. This is closely linked to various mutations in the Spike protein, some of which are shared with other variants of concern, while others are unique to Omicron. These mutations are associated with increased infectivity. We studied the effects of the Omicron variant on two key features: the level of the inflammatory response (CSS) and the emergency myelopoiesis (expansion of neutrophils and macrophages) triggered by other variants (45). To do this, we injected either S1WT or S1 Omicron into the HBV of 48 hpf larvae. We observed a decreased recruitment of neutrophils and macrophages to the injection site in the S1 Omicron group compared to the S1WT group at 6, 12, and 24 hpi (**Figures 1A, B**). Furthermore, the Omicron variant failed to induce emergency hematopoiesis at all analyzed time points, another similarity to the Delta variant (**Figures 1A, B**) (45). We next analyzed the inflammatory responses triggered by the S1WT and S1 Omicron variants of SARS-CoV-2. The Nfkb reporter activity was examined at 6, 12, and 24 hpi, alongside the inflammatory gene expression at 12 hpi. Omicron exhibited elevated levels of the Nfkb activity compared to S1WT, indicating a higher degree of inflammation at all time points (**Figure 1C**). Similarly, the transcript levels of *il1b*, *cxcl8a*, and *tnfa* were higher in S1 Omicron than in S1WT group mainly locally (**Figures 2A–C**). Additionally, the mRNA levels of *nfkb1* were higher both locally and systemically in S1 Omicron-injected larvae than in S1WT (**Figures 2D**). However, the transcript levels of the anti-inflammatory cytokine *il10* remained unchanged both locally and systemically between the S1WT and S1 Omicron groups (**Figure 2E**). Finally, caspase-1 activity levels were also higher both locally in the head and systemically of S1 Omicron-injected larvae than in S1WT-injected larvae (**Figure 2F**). Therefore, these findings suggest that the Omicron variant of SARS-CoV-2 exhibits mixed properties characteristic of both the Delta and Gamma variants (45).

### Omicron S1 variant promotes cell death of neutrophils

**Figure 3:**
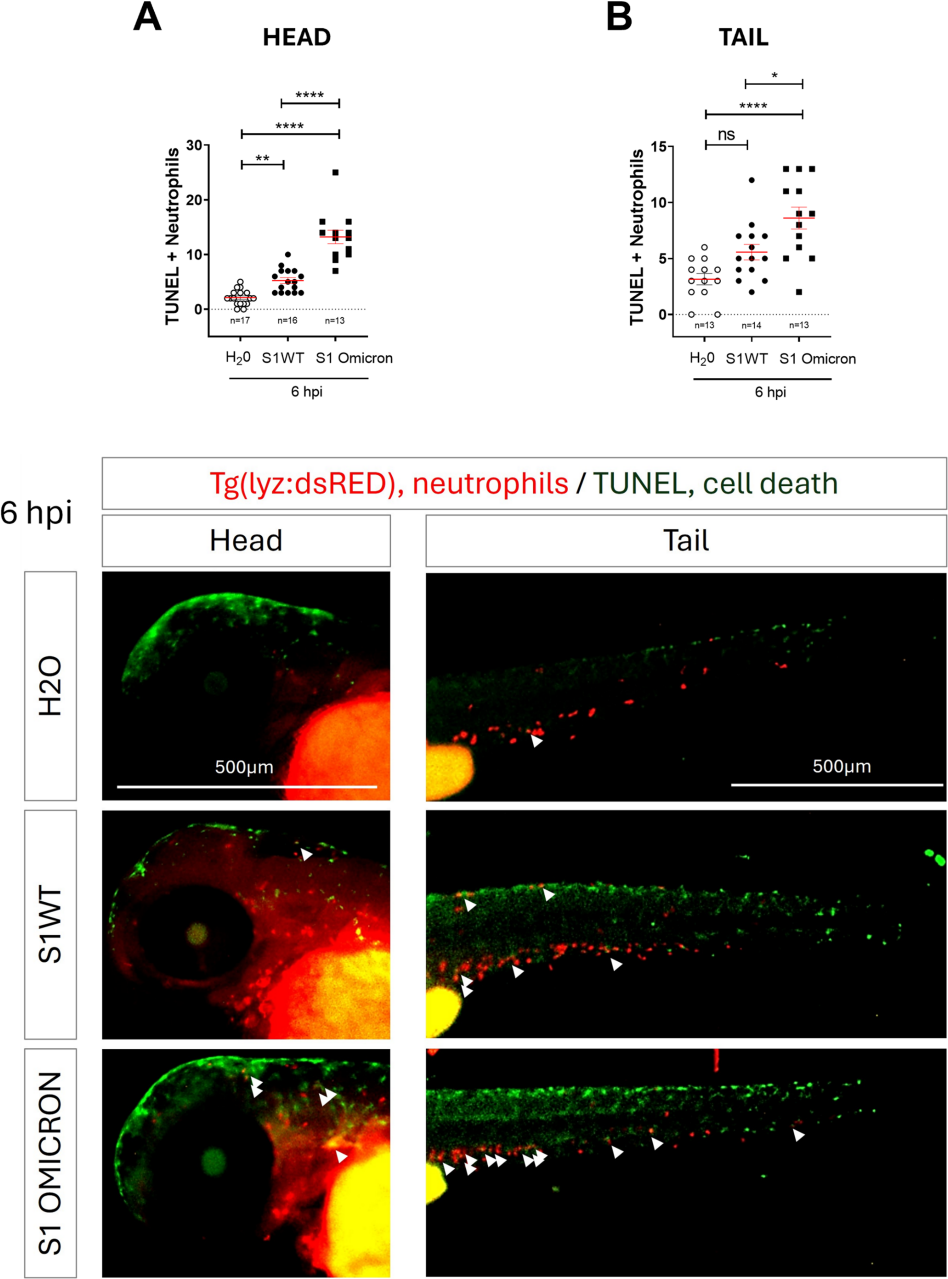
Omicron causes higher neutrophil cell death than the ancestral variant. Recombinant S1WT, S1 Omicron or vehicle (-) were injected in the hindbrain ventricle (HBV) of *Tg(lyz:DsRED)***(A, B)** of 2-dpf larvae. Tunel positive neutrophil number (double positive) was counted at 6 hpi in the head and tail of the larvae **(A, B)**. Representative photos for each treatment are shown. Scale bar 500 μm. Each dot represents one individual, and the means ± SEM for each group is also shown. *P* values were calculated using one-way analysis of variance (ANOVA) and Tukey multiple range test. ns, not significant; *P ≤ 0.05, **P ≤ 0.01 and ****p < 0.0001.

Given the high proinflammatory activity of S1 Omicron variants and the paradoxical inability to promote the expansion of neutrophils and macrophages, we sought to investigate the underlying mechanism. Increasing evidence indicates that SARS-CoV-2-induced cell death substantially contributes to viral pathogenicity and disease progression. In fact, elevated levels of pyroptosis in neutrophils and macrophages have been reported in COVID-19 patients (51–55). To determine whether the Omicron variant promotes neutrophil cell death more effectively than the wild-type strain, we quantified the number of dying neutrophils in the head and tail regions of S1-injected larvae at 6 hpi. We observed that although S1WT injection resulted in a slight increase in neutrophil cell death in the injection site, S1 Omicron markedly elevated neutrophil cell death in the injection site and to some extent in the caudal hematopoietic tissue (**Figures 3A, B**). These findings suggest that the Omicron variant possesses an enhanced capacity to induce neutrophil cell death.

### S1/S2WT trimer is not proinflammatory

**Figure 4:**
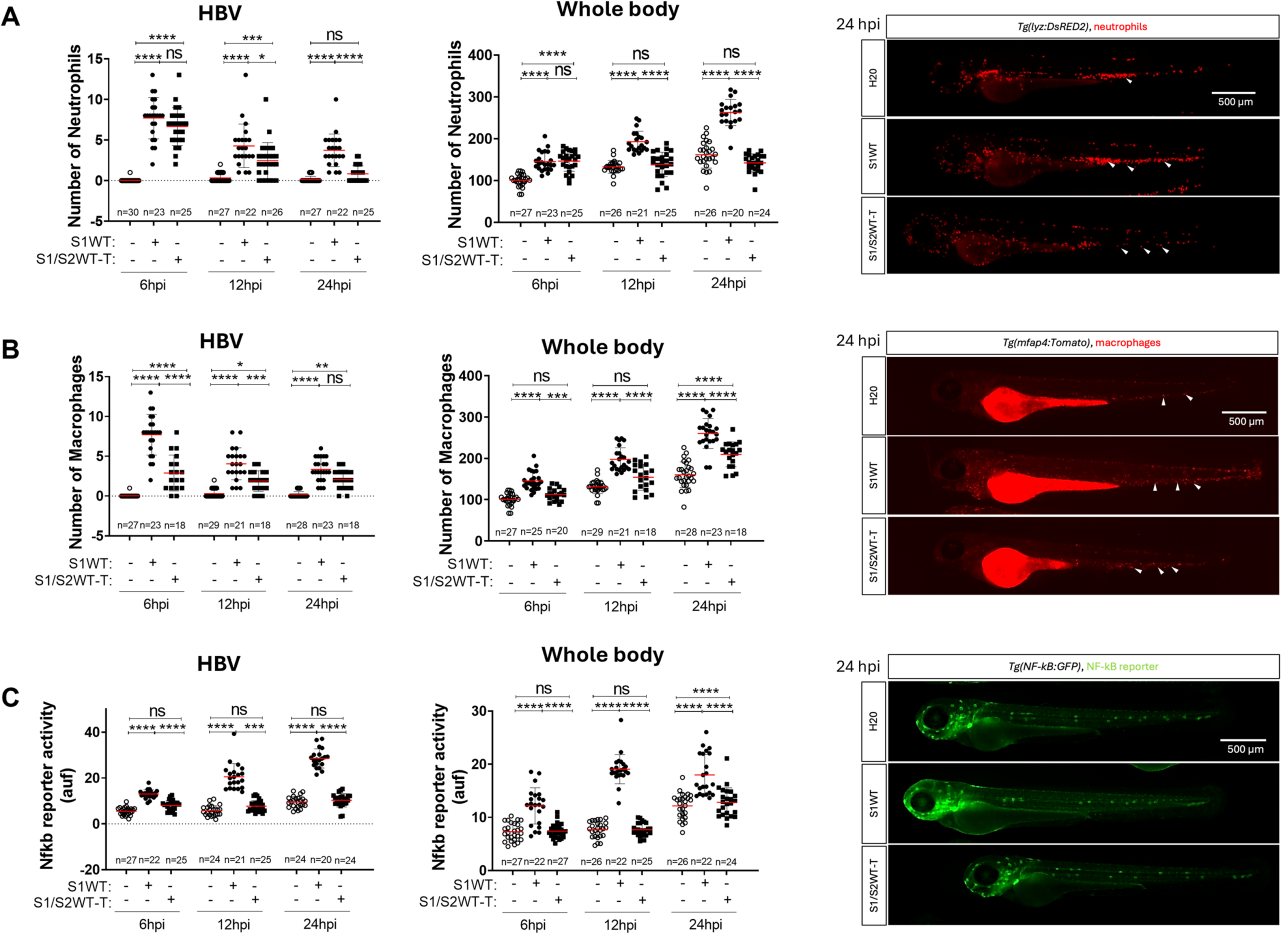
Trimeric ancestral variant induces a weaker immune response than its monomeric form. Recombinant S1WT (monomeric), S1/S2WT-T (trimeric) or vehicle (-) were injected in the hindbrain ventricle (HBV) of *Tg(lyz:DsRED)***(A)**, *Tg(mfap4:Tomato)* **(B)**, *Tg(*nfkb*:eGFP)* **(C)** 2-day postfertilization larvae (dpf). Neutrophil **(A)** and macrophage **(B)** recruitment and number and Nfkb reporter activity **(C)** were analyzed at 6, 12, and/or 24 hpi by fluorescence microscopy. Representative photos for each treatment are shown from 24 hpi. Scale bar 500 μm. Each dot represents one individual, and the means ± SEM for each group is also shown. *P* values were calculated using one-way analysis of variance (ANOVA) and Tukey multiple range test. ns, not significant; *P ≤ 0.05, **P ≤ 0.01, ***P ≤ 0.001 and ****p < 0.0001. auf, arbitrary units of fluorescence.

**Figure 5:**
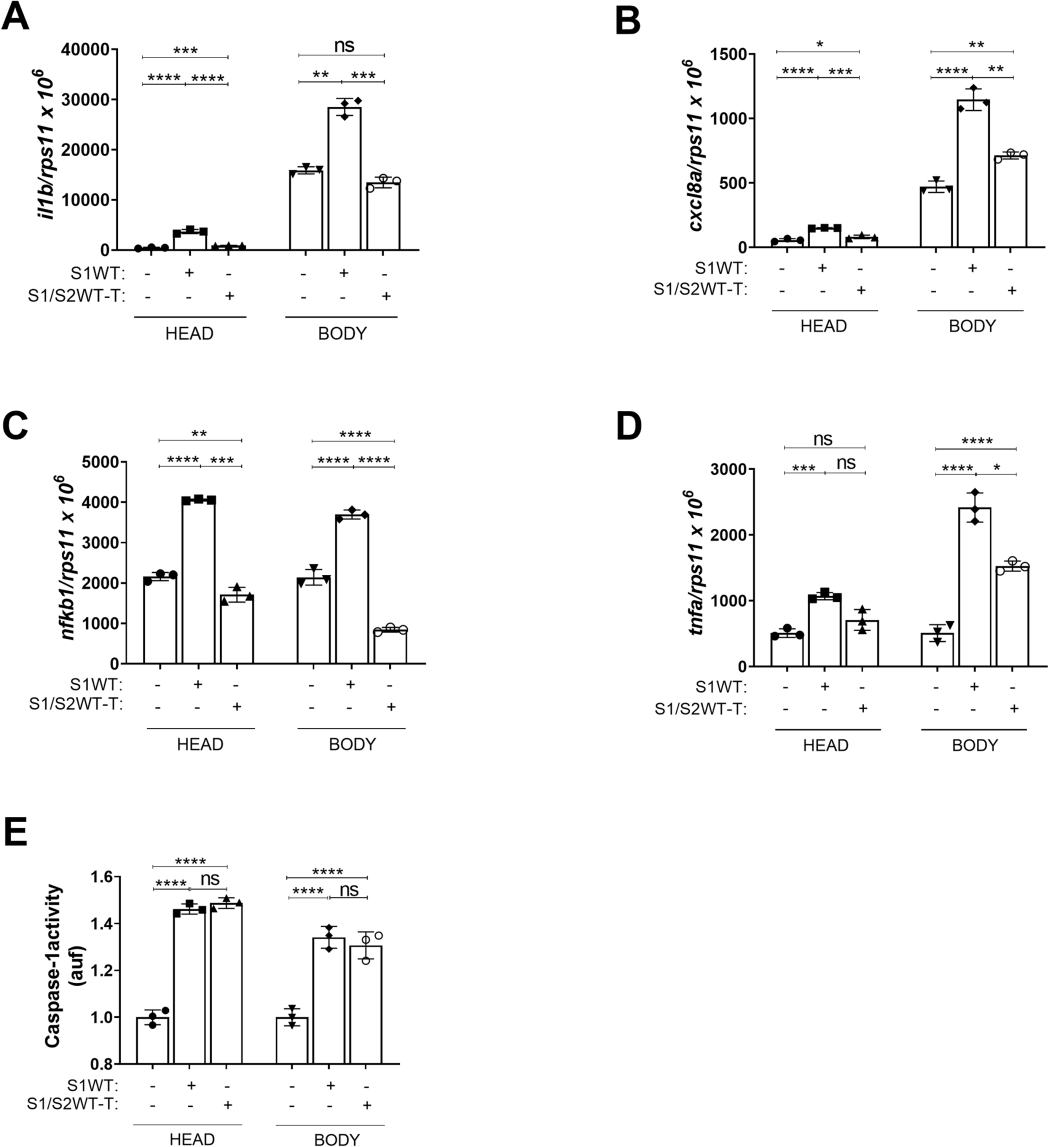
Trimeric ancestral variant induces a weaker proinflammatory response than its monomeric form. Recombinant S1WT, S1/S2WT-T or vehicle (-) were injected in the hindbrain ventricle (HBV) of WT **(A–E)** 2-day postfertilization larvae (dpf). The transcript levels of the indicated genes **(A–D)** were analyzed at 12 hpi by RT-qPCR in larval head and rest of the body and caspase-1 activity was determined at 24 hpi using a fluorogenic substrate **(E)**. Graphs shown are representative of three independent experiments; technical replicates are displayed in each graph. The means ± SEM for each group is shown. *P* values were calculated using one-way analysis of variance (ANOVA) and Tukey multiple range test. n=40 in A-D, n=35 in E. ns, not significant; *P ≤ 0.05, **P ≤ 0.01, ***P ≤ 0.001 and ****p < 0.0001. auf, arbitrary units of fluorescence.

It has previously been shown that monomeric S1WT triggers an immune response and activates emergency hematopoiesis *in vivo* in zebrafish (45). To check whether the 3D conformation of the S1 viral protein influences receptor recognition and elicits a different immune response, we injected the trimeric form of S1WT combined with S2 domain (S1WT-T) into the HBV of 48 hpf zebrafish larvae. Then, the phagocyte recruitment to the injection site, their total numbers in the whole body, and the inflammation patterns at 6, 12, and/or 24 hpi were analyzed. Importantly, our results revealed that S1/2WT-T had a different effect on neutrophil and macrophage recruitment compared to the monomeric S1WT. Thus, fewer neutrophils and macrophages were recruited to HBV following S1/2WT-T injection, with this difference being more prominent at later time points (**Figures 4A, B**). Furthermore, S1/S2WT-T injection only modestly increased the total number of phagocytes in the whole body, in contrast to the significant increase observed in S1WT-injected animals (**Figures 4A, B**). In terms of inflammation, the Nfkb response was reduced in S1/S2WT-T-injected larvae at the injection site as well as in the whole body, compared to the monomeric S1WT (**Figure 4C**). To confirm that, we measured the transcript levels of several pro-inflammatory molecules at 12 hpi in the head (locally) and the body/tail (systemically), finding that the transcript levels of *il1b*, *cxcl8a*, *nfkb1* and *tnfa* hardly increased locally and systemically in S1/S2WT-T-injected larvae (**Figures 5A–D**). Nevertheless, caspase-1 activity was induced at similar levels by S1WT and S1/S2WT-T. (**Figure 5E**). These results demonstrate that S1/S2WT-T fails to elicit inflammation and emergency hematopoiesis despite activating the inflammasome to the same extent as the monomeric form of S1WT.

### Ace2 plays a dual role in regulating the COVID-19-associated CSS triggered by the Spike protein

**Figure 6:**
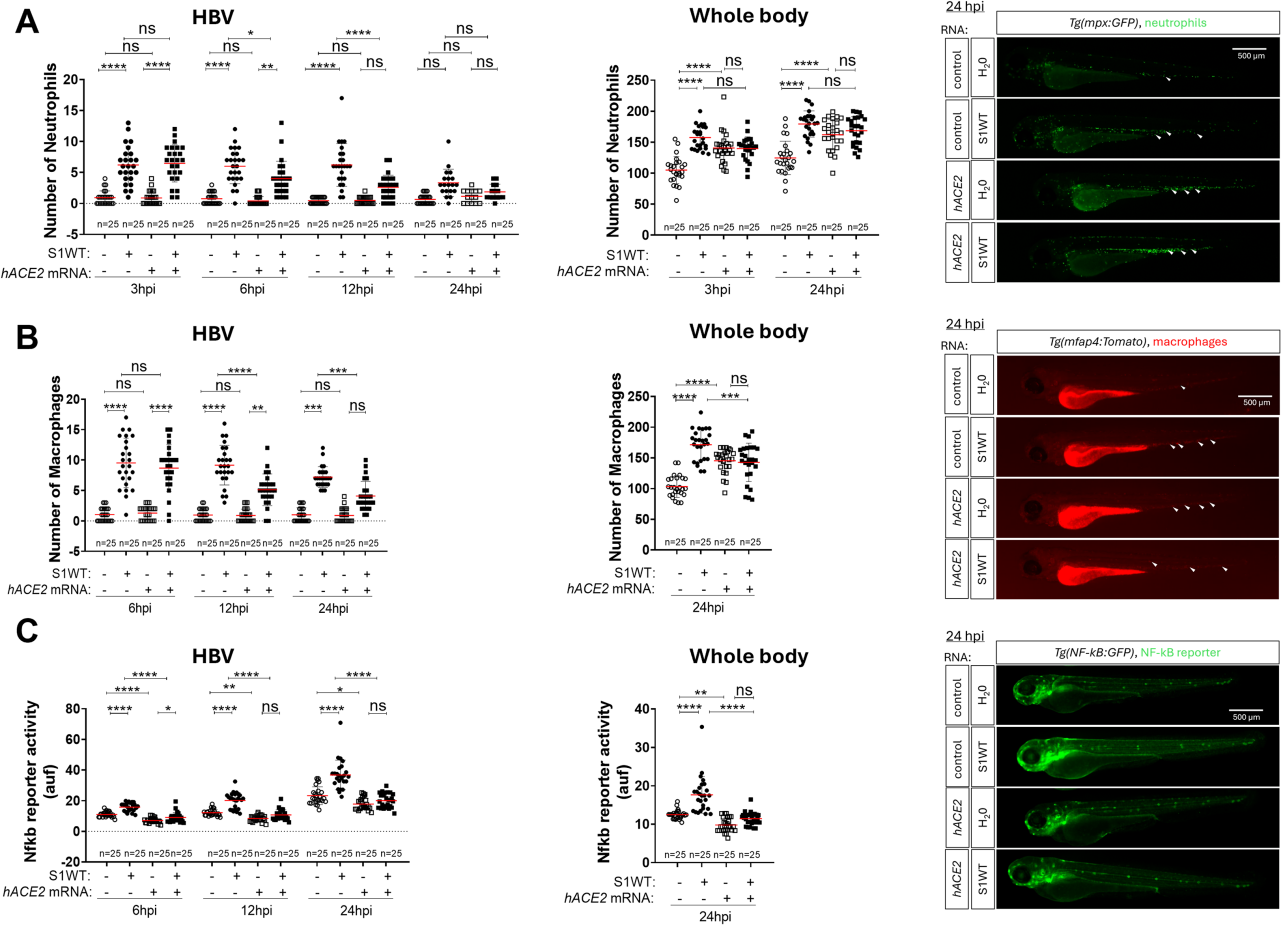
Human ACE2 promotes the resolution of inflammation driven by the monomeric ancestral variant. One-cell-stage zebrafish eggs of *Tg(mpx:eGFP)***(A)**, *Tg(mfap4:Tomato)* **(B)** and *Tg(*nfkb*:eGFP)* **(C)** larvae were microinjected with control or human *ACE2* mRNA. Recombinant S1WT or vehicle (-) were injected in the hindbrain ventricle (HBV) of 2-dpf larvae. Neutrophil **(A)** and macrophage **(B)** recruitment and number and Nfkb activation **(C)** were analyzed at 3, 6, 12, and/or 24 hpi by fluorescence microscopy. Representative photos for each treatment are shown from 24 hpi. Scale bar 500 μm. Each dot represents one individual, and the means ± SEM for each group is also shown. *P* values were calculated using one-way analysis of variance (ANOVA) and Tukey multiple range test. ns, not significant; *P ≤ 0.05, **P ≤ 0.01, ***P ≤ 0.001 and ****p < 0.0001. auf, arbitrary units of fluorescence.

**Figure 7:**
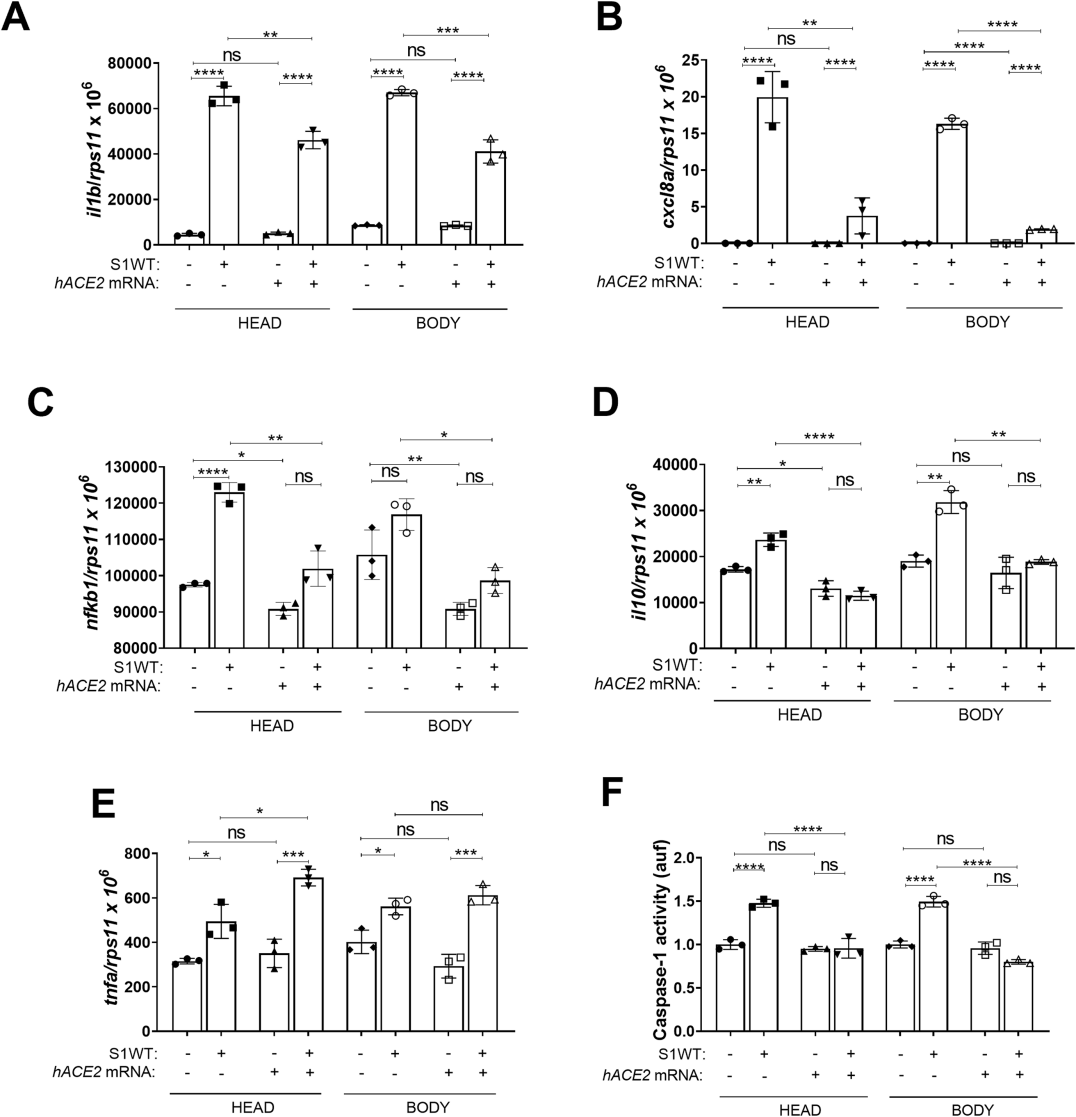
Human ACE2 attenuates the inflammation driven by the monomeric ancestral variant. One-cell-stage zebrafish eggs of WT **(A–F)** larvae were microinjected with control or human *ACE2* mRNA. Recombinant S1WT or vehicle (-) were injected in the hindbrain ventricle (HBV) of 2-dpf larvae. The transcript levels of the indicated genes **(A–E)** were analyzed at 12 hpi by RT-qPCR in larval head and rest of the body and caspase-1 activity was determined at 24 hpi using a fluorogenic substrate **(F)**. Graphs shown are representative of three independent experiments; technical replicates are displayed in each graph. The means ± SEM for each group is shown. *P* values were calculated using one-way analysis of variance (ANOVA) and Tukey multiple range test. n=45 in **(A–E)**, n=45 in **(F)**. ns, not significant; *P ≤ 0.05, **P ≤ 0.01, ***P ≤ 0.001 and ****p < 0.0001. auf, arbitrary units of fluorescence.

We have previously demonstrated that endogenous zebrafish Ace2 attenuated the COVID-19-associated CSS triggered by monomeric S1 through the production of angiotensin (1-7) (45). We decided to investigate whether hACE2 overexpression from the earliest stages of development was also able to modulate the zebrafish immune response to S1WT. For this, we injected the mRNA of *hACE2* into one-cell-stage embryos, which did not result in any observable morphological defects or developmental abnormalities, nor did it affect larval survival at 24 hours post-fertilization (hpf) (**Supplementary Figures S1A, B**). However, hACE2-overexpressing embryos exhibited accelerated development compared to controls (**Supplementary Figure S1C**). The phagocyte recruitment experiments revealed that while hACE2 overexpression did not alter the early-phase recruitment of neutrophils and macrophages (**Figures 6A, B**), it robustly reduced the number of recruited cells at longer time points, suggesting that it promoted inflammation resolution (**Figures 6A, B**). Additionally, a slight increase in the total number of neutrophils and macrophages in the whole body of hACE2 overexpressing larvae was observed, potentially due to the accelerated development associated with hACE2 overexpression (**Figures 6A, B**, **Supplementary Figures S1D, S1E**). Although no significant differences were detected in S1WT-induced neutrophil expansion between control and hACE2-overexpressing larvae, the expansion of macrophages promoted by S1WT was attenuated by hACE2 (**Figures 6A, B**). Interestingly, hACE2 overexpression resulted in reduced Nfkb reporter activity in both control and S1WT-injected larvae (**Figure 6C** and **Supplementary Figure S1F**). Similarly, forced expression of hACE2 impaired the S1WT-mediated induction of the transcript levels of *il1b, cxcl8a, nfkb1* and *il10* (**Figures 7A–D**), while those of *tnfa* were mostly unaffected by hACE2 (**Figure 7E**). Notably, caspase-1 activity was attenuated upon S1WT injection (**Figure 7F**).

**Figure 8:**
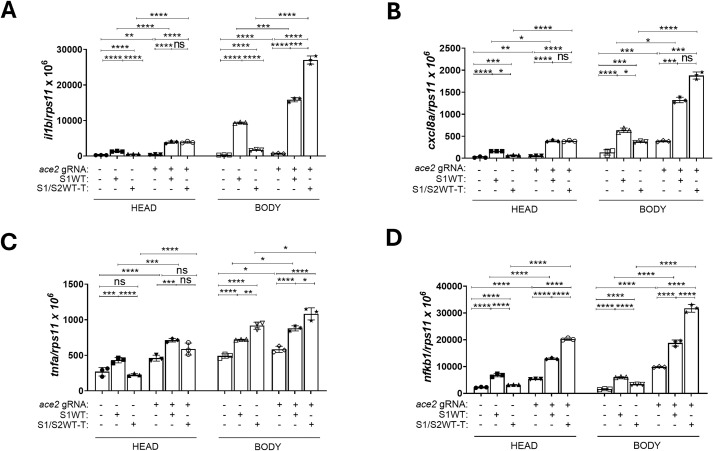
Zebrafish Ace2 neutralizes trimeric ancestral variant but not its monomeric form. One-cell-stage zebrafish eggs of WT were microinjected with control or *ace2* gRNA-Cas9 complexes **(A–D)**. Recombinant S1WT, S1WT-T or vehicle (-) were injected in the hindbrain ventricle (HBV) of these 2-dpf larvae. The transcript levels of the indicated genes **(A–D)** were analyzed at 12 hpi by RT-qPCR in larval head and rest of the body. Graphs shown are representative of three independent experiments; technical replicates are displayed in each graph. The means ± SEM for each group is shown. *P* values were calculated using one-way analysis of variance (ANOVA) and Tukey multiple range test. n=45 in **(A–D)**. ns, not significant; *P ≤ 0.05, **P ≤ 0.01, ***P ≤ 0.001 and ****p < 0.0001.

Although all the above findings support that both zebrafish and human ACE2 dampen COVID-19-associated CSS driven by the Spike protein via the production of Ang-(1-7), they failed to explain the reduced proinflammatory activity of the trimeric S compared with its monomeric form. We, therefore, hypothesized that the trimeric form of S1/S2WT-T is bound and neutralized by endogenous zebrafish Ace2, while monomeric S1WT cannot be neutralized by Ace2. To test this hypothesis, we injected S1/S2WT-T into Ace2-deficient larvae, generated by CRISPR-Cas9 technology, and then assessed the transcript levels of proinflammatory genes at 12 hpi using RT-qPCR. The efficiency of the Ace2 CRISPR was confirmed to be over 70% using the TIDE web tool (**Supplementary Figure S2**). Our results showed that S1/S2WT-T was even more proinflammatory than monomeric S1WT in Ace2-deficient larvae (**Figures 8A–D**). These results suggest that endogenous ACE2 plays a dual role in the regulation of COVID-19-associated CSS driven by SARS-CoV-2 Spike protein and its derived fragments.

## Discussion

The Omicron variant from SARS-CoV-2 is characterized by numerous mutations in its spike protein, particularly in the receptor-binding domain, leading to increased transmissibility and reduced antibody and vaccine effectiveness. These changes result in distinct biological behavior compared to earlier variants, including altered immune responses and pathogenicity. Studying different variants individually is essential, as each may differ in how it spreads, evades immunity, and triggers inflammation. Understanding these differences is key for developing effective diagnostics, treatments, and public health strategies (56, 57). Our *in vivo* findings indicate that the Omicron variant of SARS-CoV-2 elicits a distinct immune response compared to ancestral variants. In contrast to the S1WT protein, S1 Omicron induced significantly lower recruitment and expansion of neutrophils and macrophages at all examined time points. Despite this attenuated cellular response, Omicron triggered a more pronounced systemic inflammatory response, characterized by elevated Nfkb activity and increased expression of selected pro-inflammatory cytokines, particularly in the inflammatory site. Notably, S1 Omicron induced stronger caspase-1 activation both locally and systemically than S1WT, suggesting variant-specific activation of the inflammasome. These results imply that S1 Omicron may promote inflammation via alternative immune pathways, with a prominent role for inflammasome activation. This observation is supported by *in vitro* experiments in human monocytic THP-1 cells, where Omicron variant infection led to the highest IL-1β secretion (58). Enhanced inflammasome activity may contribute to more efficient viral neutralization and could partially account for the observed attenuation in clinical severity (58).

Emerging evidence suggests that the Omicron variant modulates neutrophil cell death through mechanisms distinct from those of earlier SARS-CoV-2 variants. Neutrophils are essential components of the innate immune system, and their activation followed by cell death via apoptosis, pyroptosis, or NETosis, plays a pivotal role in regulating inflammation and mediating pathogen clearance (59–61). Despite the generally milder respiratory manifestations associated with Omicron infections, our findings demonstrate that this variant induced substantial neutrophil cytotoxicity. This increased cell death, likely driven by enhanced inflammasome activation, may contribute to the lower virulence observed with Omicron compared to the wild-type strain and other variants of concern. Notably, our data also shows similar kinetics in macrophage dynamics, supporting the hypothesis of a shared inflammasome-dependent mechanism. These observations are consistent with previous reports indicating that Omicron can induce inflammasome-mediated death in both neutrophils and macrophages (51–53, 55, 62). Further studies are warranted to elucidate the molecular pathways through which Omicron influences myeloid cell survival and death, which could uncover novel therapeutic targets for modulating host responses in SARS-CoV-2 infection.

Although previous *in vitro* studies have demonstrated the high interaction and affinity between the SARS-CoV-2 S protein and the human ACE2 receptor, unfortunately a more comprehensive understanding of the dynamic immune response, the receptor engagement, and the downstream signaling pathways activated following such interaction cannot be fully captured in cell culture systems (63, 64). The zebrafish model offers a unique opportunity to dissect the complex interactions between viral proteins and host receptors *in vivo*, shedding light on the role of ACE2 in modulating the immune response and providing insights into the broader implications of ACE2-mediated signaling in COVID-19 pathogenesis (45, 46, 65). In fact, we have previously reported that the zebrafish Ace2 plays a conserved anti-inflammatory role in modulating COVID-19-associated CSS via the production of Ang-(1-7) (45). We confirm in the present study the evolutionary conservation of this axis by demonstrating that overexpression of human ACE2 in zebrafish larvae resulted in accelerated resolution of the inflammation upon S1WT injection, likely through the production of Ang-(1-7), since endogenous zebrafish Ace2 failed to neutralize monomeric S1WT. Specifically, one of the most interesting results of our study is that the trimeric S1/S2WT hardly induced the recruitment of neutrophils and macrophages at the injection site and failed to promote a systemic inflammatory response, while genetic inhibition of zebrafish Ace2 fully restored its hyperinflammatory activity. This is consistent with the 100-fold lower affinity of monomeric S1 compared with trimer S1/S2 (EC_50_ of 300–700 and 4–24 ng/mL, respectively) (https://www.sinobiological.com/recombinant-proteins/2019-ncov-cov-spike-40591-v08b1, https://www.sinobiological.com/recombinant-proteins/sars-cov-2-cov-spike-40589-v08h8). The attenuated phagocyte recruitment and diminished inflammatory gene expression observed in response to the trimeric form are consistent with patterns described in other viral infections, where the structural integrity of viral proteins critically influences immune activation and may facilitate immune evasion mechanisms (20, 66, 67).

Emphasizing the importance of our data, although the SARS-CoV-2 spike protein is initially present in a trimeric conformation on the viral surface, it undergoes proteolytic fragmentation during infection, and monomeric spike fragments have been detected in the bloodstream of infected patients, persisting even several months after recovery (22–24). Moreover, recombinant vaccines comprising residues 319–545 of the RBD of the S protein have also been used to elicit robust functional antibody responses in mice, rabbits, and non-human primates (21). Furthermore, this also aligns with other previously published results revealing that trimeric S1 serves as a potent inhibitor of SARS-CoV-2 infection, exhibiting affinity for ACE2 and effectively blocking viral entry more efficiently than monomeric RBD (68). These results confirm other anti-inflammatory role for ACE2 in direct neutralizing of the trimeric fragments of Spike leading to diminish of the inflammatory effects that are seen with monomeric forms of S protein. Therefore, all these findings together emphasize the complexity of the host-pathogen interactions in viral infections and provide potential targets for future therapeutic strategies for COVID-19, including the design of vaccines and the development of novel strategies to prevent SARS-CoV-2 infection from spreading to neighboring cells and, therefore, to control viral dissemination within the host.

**Figure 9:**
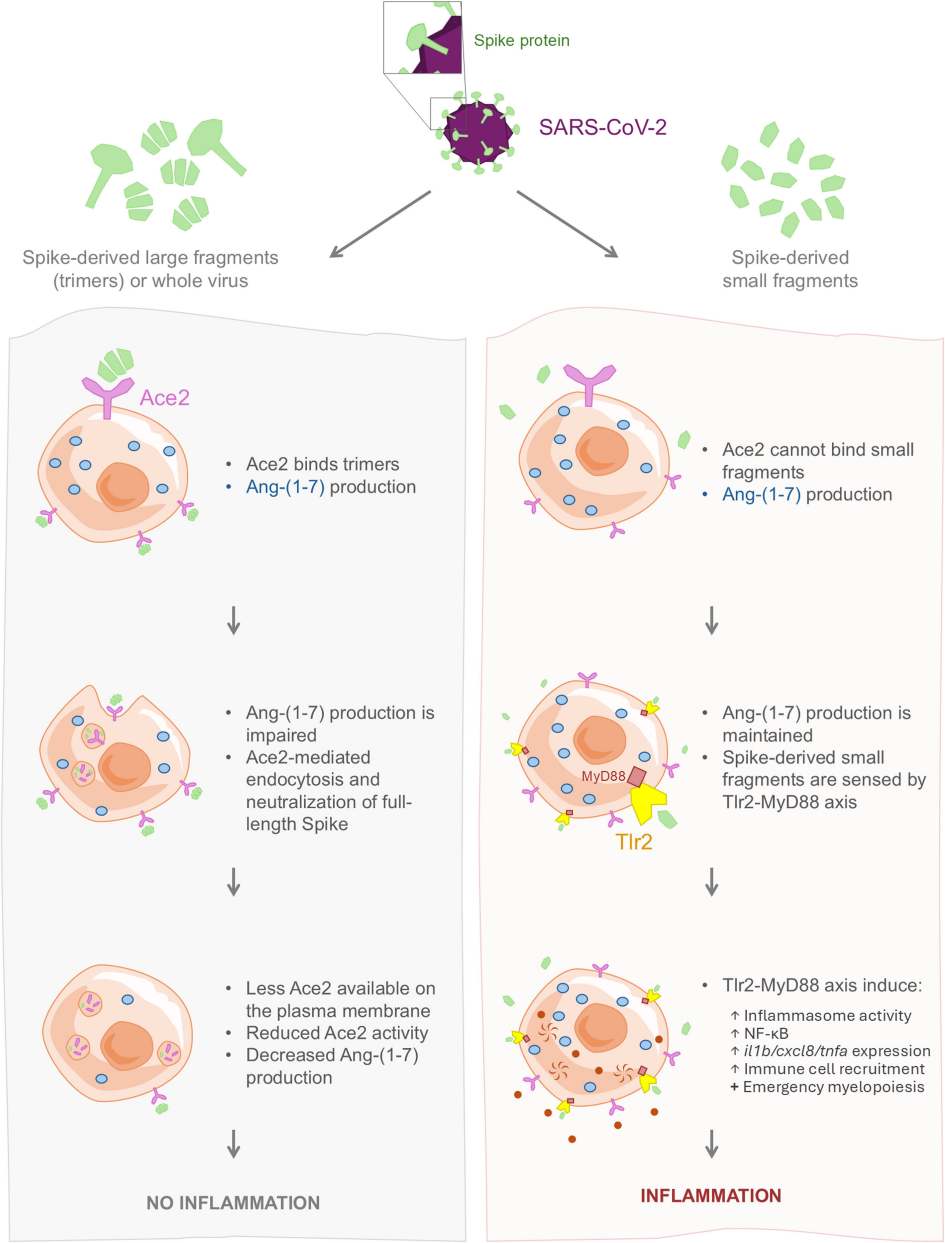
Schematic representation of the dual effect of Ace2 in regulating COVID-19-associated CSS. Ace2 produces Ang-(1-7) during viral infection to dampen inflammation. In addition, Ace2 is also able to bind and neutralize trimeric Spike proteins but not its monomeric derived fragments generated during the infection. Monomeric Spike-derived fragments activate Tlr2 and inflammasome signaling pathways to contribute to the CSS. .

In summary, our study reveals that the S1 domain of the Omicron variant triggers a more robust proinflammatory response than the ancestral S1WT, primarily through enhanced inflammasome activation and promotion of neutrophil and macrophage death. We further demonstrate that both the trimeric full-length Spike protein and its monomeric fragments—generated during infection—contribute to the COVID-19-associated CSS, likely via TLR2 and inflammasome activation (**Figure 9**). Importantly, we identify a dual anti-inflammatory role for ACE2 in SARS-CoV-2 pathogenesis: (1) enzymatic production of Ang-(1–7), and (2) direct neutralization of trimeric Spike, thereby preventing immune overactivation. These findings highlight the differential immunostimulatory potential of Spike-derived fragments and underscore the importance of dissecting their interactions with host immune pathways. A deeper understanding of these dynamics is essential to clarify their roles in both acute disease and post-acute sequelae (long COVID) and may inform the development of targeted therapeutic strategies aimed at modulating inflammasome activity and ACE2-mediated regulation.

## Materials and methods

### Animals

Zebrafish (*Danio rerio* H.) were obtained from the Zebrafish International Resource Center and mated, staged, raised, and processed as described (69). The zebrafish lines Tg(*mpx:eGFP*)^i114^ (70), Tg(*lyz:DsRED2*)^nz50^ (71), Tg(*mfap4.1:Tomato*)^xt12^ (72), Tg(*NFkB-RE:eGFP*)^sh235^ referred to as nfkb:eGFP (73), and casper (mitfa^w2/w2^; mpv17^a9/a9^) (74) were previously described. The experiments performed comply with the Guidelines of the European Union Council (Directive 2010/63/EU) and the Spanish RD 53/2013. The experiments and procedures performed were approved by the Bioethical Committees of the University of Murcia (approval number #669/2020).

### DNA constructs

The human *ACE2* gene (accession number ENSG00000130234) was optimized for zebrafish codon usage and synthesized by IDT.

### Analysis of gene expression

Total RNA was extracted from 40–45 head/tail part of the zebrafish body with TRIzol reagent (Invitrogen) following the manufacturer’s instructions and treated with DNase I, amplification grade (1 U/mg RNA: Invitrogen). SuperScript IV RNase H Reverse Transcriptase (Invitrogen) was used to synthesize first-strand cDNA with random primer from 1mg of total RNA at 50°C for 50 min. Real-time PCR was performed with an ABIPRISM 7500 instrument (Applied Biosystems) using SYBR Green PCR Core Reagents (Applied Biosystems). Reaction mixtures were incubated for 10 min at 95°C, followed by 40 cycles of 15 s at 95°C, 1 min at 60°C, and finally 15 s at 95°C, 1 min 60°C, and 15 s at 95°C. For each mRNA, gene expression was normalized to the ribosomal protein S11 (*rps11*) content in each sample using the Pfaffl method (75). The primers used are shown in **Supplementary Table S1**. In all cases, each PCR was performed with triplicate samples and repeated at least with two independent samples.

### Crispr, RNA and recombinant protein injections in zebrafish

crRNA for zfAce2 (45) and tracrRNA were resuspended in Nuclease-Free Duplex Buffer to 100 µM. 1µl of each was mixed and incubated for 5 min at 95°C for duplexing. After removing from the heat and cooling to room temperature, 1.43 µl of Nuclease-Free Duplex Buffer was added to the duplex, giving a final concentration of 1000 ng/µl. Finally, the injection mix was prepared by mixing 1 µl of duplex, 2.55 µl of Nuclease-Free Duplex Buffer, 0.25 µl Cas9 Nuclease V3 (IDT, 1081058) and 0.25 µl of phenol red, giving final concentrations of 250 ng/µl of gRNA duplex and 500 ng/µl of Cas9. The prepared mix was microinjected into the yolk sac of one- to eight-cell-stage embryos using a microinjector (Narishige) (0.5–1 nl per embryo). The same amounts of gRNA were used in all the experimental groups. The efficiency of gRNA was checked by amplifying the target sequence with a specific pair of primers (**Supplementary Table S2**) and the TIDE webtool (https://tide.nki.nl/) (76).

Recombinant His-tagged Spike S1 wild-type (#40591-V08B1), S1 Omicron (#40592-V08H121) or Spike S1/S2 TRIMER wild-type (#40589-V08H8), all from Sino Biological at a concentration of 0.25 mg/ml supplemented with phenol red were injected into the hindbrain (1 nl) of 48 hpf zebrafish larvae.

*In vitro*-transcribed RNA was obtained following manufacturer’s instructions (mMESSAGE mMACHINE kit, Ambion). RNA of hACE2 was mixed in microinjection buffer and microinjected into the yolk sac of one-cell-stage embryos using a microinjector (Narishige; 0.5–1 nl per embryo). The same amount of RNA was used in all experimental groups.

### Chemical treatments

In some experiments, 24 hpf embryos were treated with 0.3% N-Phenylthiourea (PTU, Sigma-Aldrich) to inhibit melanogenesis.

### Caspase-1 activity assay

The caspase-1 activity was determined with the fluorometric substrate Z-YVAD 7-Amido-4-trifluoromethylcoumarin (Z-YVAD-AFC, caspase-1 substrate VI, Calbiochem) as described previously (46, 77). In brief, 35–45 heads or the rest of the bodies were lysed in hypotonic cell lysis buffer (25 mM 4-(2-hydroxyethyl) piperazine-1-ethanesulfonic acid, 5 mM ethylene glycol-bis(2-aminoethylether)-N,N,N´,N´-tetraacetic acid, 5 mM dithiothreitol, 1:20 protease inhibitor cocktail (Sigma-Aldrich), pH 7.5) on ice for 10 min. For each reaction, 50 mg protein were incubated for 90 min at room temperature with 50 mM YVAD-AFC and 50 ml of reaction buffer (0.2% 3-[(3-cholamidopropyl)dimethylammonio]-1-propanesulfonate (CHAPS), 0.2 M 4-(2-hydroxyethyl) piperazine-1-ethanesulfonicacid, 20% sucrose, 29 mM dithiothreitol, pH 7.5). After the incubation, the fluorescence of the AFC released from the Z-YVAD-AFC substrate was measured with a FLUOstart spectofluorometer (BGM, LabTechnologies) at an excitation wavelength of 405 nm and an emission wavelength of 492 nm. A representative caspase-1 activity graph out of three repeats is shown in figures.

### *In-vivo* imaging

To study immune cell recruitment to the injection site and Nfkb activation, 2 dpf Tg(*mpx:eGFP*), Tg(*mfap4:tomato*) or Tg(*nfkb:egfp*) larvae were anaesthetized in embryo medium with 0.16 mg/ml tricaine. Images of the hindbrain, head or the whole-body area were taken 3, 6, 12 and 24 h post-injection (hpi) using a Leica MZ16F fluorescence stereomicroscope. The number of neutrophils or macrophages was determined by counting visually and the fluorescence intensity was obtained and analyzed with ImageJ (FIJI) software (78).

### Developmental scoring

Zebrafish larvae injected with hACE2 were sorted at 2 hpf to choose the ones being at the same developmental stage and raised at similar densities. Then, at 24 hpf number of dead/alive embryos has been counted and within the survived group the number of embryos with any malformation has been counted. At 26 hpf number of otic vesicle structures that could fit between the eye and otic vesicle in each larva were estimated. The higher number of the otic vesicles fitted, lower level of the larval development estimated (79).

### Statistical analysis

Statistical analysis was performed using Prism 8.0 (GraphPad Software, CA, USA). No calculation was performed to predetermine sample size; experiments were repeated three times to ensure robustness. Embryos were randomly allocated to each experimental condition and analyzed in blind samples. No data was excluded from the analysis. Data are shown as mean ± s.e.m. and were analyzed by analysis of variance and a Tukey multiple range test to determine differences between groups. The differences between the two samples were analyzed by the two-sided Student’s *t*-test. The data met normal distribution assumption when required and showed similar variances. A log-rank test was used to calculate the statistical differences in the survival of the different experimental groups. A *p*-value <0.05 was considered statistically significant.

## Data Availability

The raw data supporting the conclusions of this article will be made available by the authors, without undue reservation.
