# Supplementary figures and images for: Dual role of ACE2 in regulating inflammation triggered by Omicron S1 and other SARS-CoV-2 Spike variants
Source: Front Immunol. 2026 Jan 6;16:1667880. doi: 10.3389/fimmu.2025.1667880 (PMC12816390; doi:10.3389/fimmu.2025.1667880)

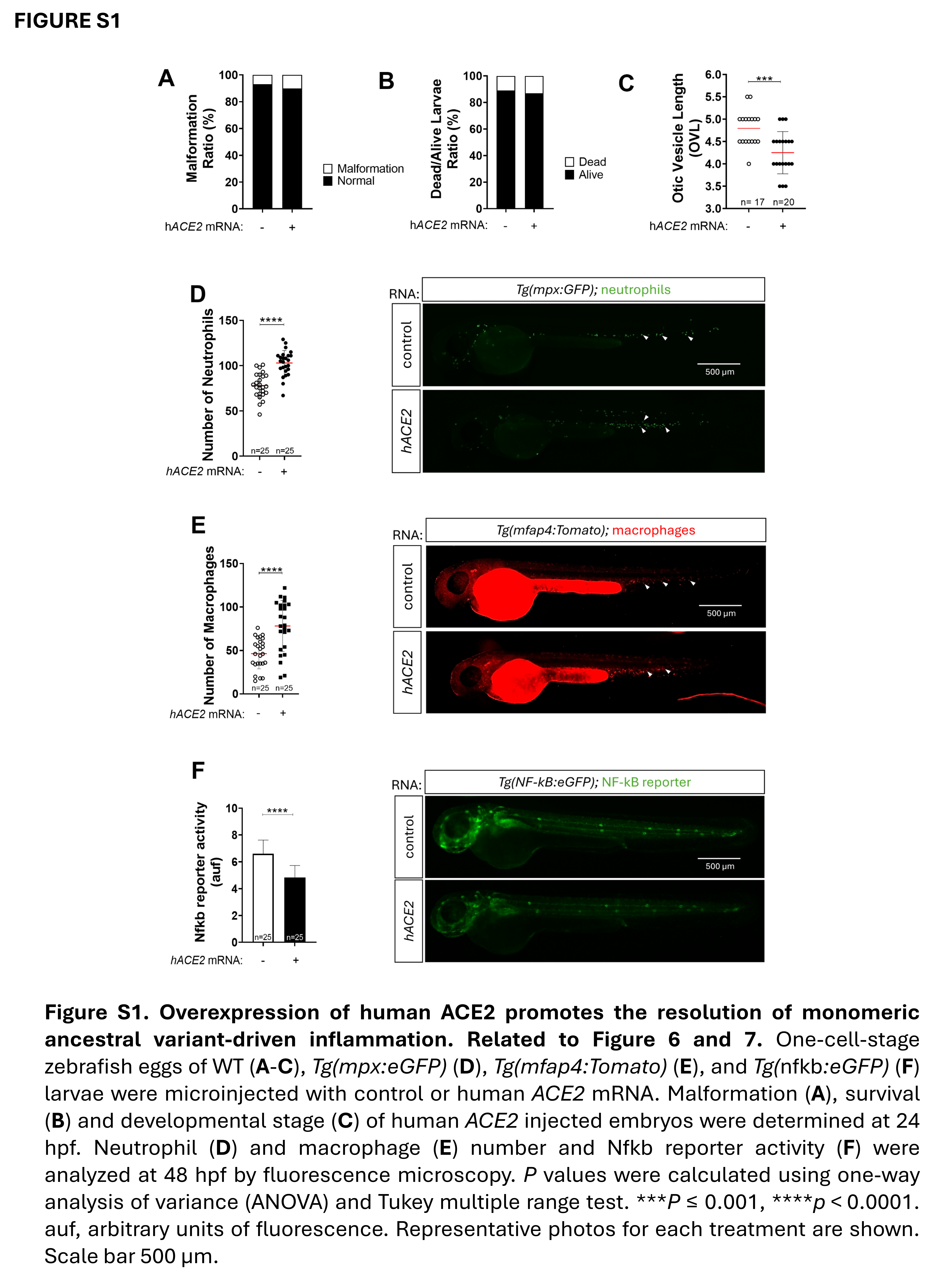

Supplement: Supplementary file 1 [file Image1.tif]

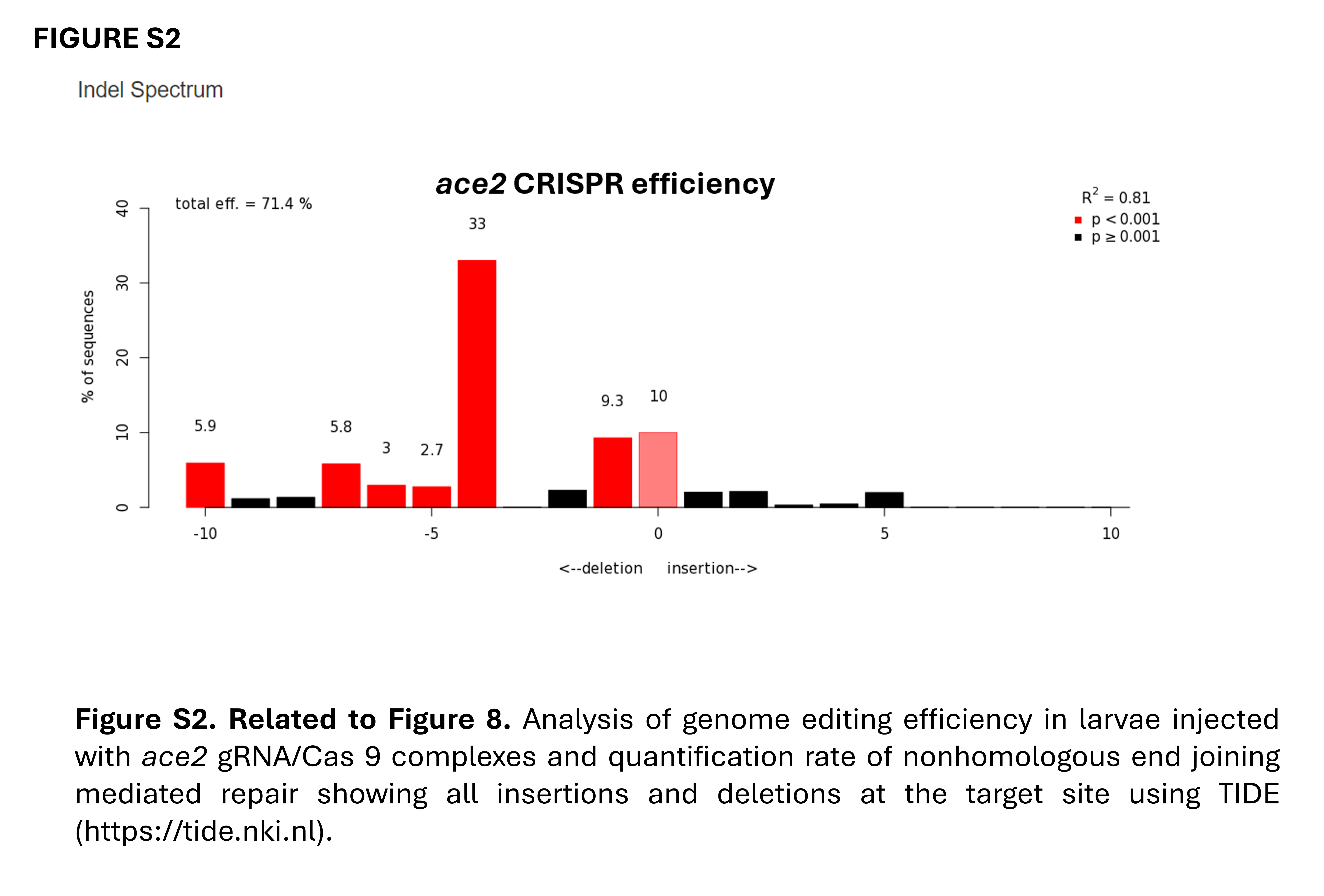

Supplement: Supplementary file 2 [file Image2.tif]
