# Supplementary material for: Dual role of ACE2 in regulating inflammation triggered by Omicron S1 and other SARS-CoV-2 Spike variants
Source: Front Immunol. 2026 Jan 6;16:1667880. doi: 10.3389/fimmu.2025.1667880 (PMC12816390; doi:10.3389/fimmu.2025.1667880)
Supplement: Supplementary file 3 [file Table1.docx]

**Table S1.** Primers used in this study. The gene symbols followed the Zebrafish Nomenclature Guidelines (http://zfin.org/zf_info/nomen.html).

| **Gene** | **ENA or ENSEMBL accession number** | **Name** | **Sequence (5’**→**3’)** | **Use** |
| --- | --- | --- | --- | --- |
| *rps11* | NM_213377.1 | F | ACAGAAATGCCCCTTCACTG | RT-qPCR |
|  |  | R | GCCTCTTCTCAAAACGGTTG |  |
| *il1b* | NM_212844.2 | F | GCCTGTGTGTTTGGGAATCT |  |
|  |  | R | TGATAAACCAACCGGGACA |  |
| *nfkb1* | ENSDARG00000105261.2 | F | TTCTTCTTGGTCACGTGCAG |  |
|  |  | R | ACTCTCAGCATCCGCATCTT |  |
| *tnfa* | NM_212859.2 | F | GCGCTTTTCTGAATCCTACG |  |
|  |  | R | TGCCCAGTCTGTCTCCTTCT |  |
| *cxcl8a* | XM_001342570.7 | F | GTCGCTGCATTGAAACAGAA |  |
|  |  | R | CTTAACCCATGGAGCAGAGG |  |
| *il10* | NM_001020785.2 | F | AACTCAAGCGGGATATGGTG |  |
|  |  | R | ATCAAGCTCCCCCATAGCTT |  |
| *ace2* | NM_001007297.1 | F | AGAGGCATATGCAATCTGGAGC | Target sequence amplification from gDNA |
|  |  | R | ATACATCTGGCTCTTTAAACTCACA |  |
