# Supplementary material for: Dual role of ACE2 in regulating inflammation triggered by Omicron S1 and other SARS-CoV-2 Spike variants
Source: Front Immunol. 2026 Jan 6;16:1667880. doi: 10.3389/fimmu.2025.1667880 (PMC12816390; doi:10.3389/fimmu.2025.1667880)
Supplement: Supplementary file 4 [file Table2.docx]

**Table S2.** gRNA used in this study. The gene symbols followed the Zebrafish Nomenclature Guidelines (http://zfin.org/zf_info/nomen.html).

| **Gene** | **Name** | **Sequence (5’**→**3’)** |
| --- | --- | --- |
| *ace2* | Dr.Cas9.ACE2.1.AC | CTCCAAAGTCTGACAATCGG |
